# Use of *traC* Gene to Type the Incidence and Distribution of pXFAS_5235 Plasmid-Bearing Strains of *Xylella fastidiosa* subsp. *fastidiosa* ST1 in Spain

**DOI:** 10.3390/plants11121562

**Published:** 2022-06-13

**Authors:** María Pilar Velasco-Amo, Luis F. Arias-Giraldo, Concepción Olivares-García, Nicolás Denancé, Marie-Agnès Jacques, Blanca B. Landa

**Affiliations:** 1 Institute for Sustainable Agriculture (IAS), Spanish National Research Council (CSIC), 14004 Córdoba, Spain; lfarias@ias.csic.es (L.F.A.-G.); colivares@ias.csic.es (C.O.-G.); 2 Groupe d’Étude et de controle des Variétes Et des Semences GEVES, CEDEX, F-49071 Beaucouzé, France; nicolas.denance@geves.fr; 3 University of Angers, Institut Agro, INRAE, IRHS, SFR QUASAV, F-49000 Angers, France; marie-agnes.jacques@inrae.fr

**Keywords:** phylogenetic analysis, plasmids, quarantine, *traC* gene, *Xylella fastidiosa*

## Abstract

*Xylella fastidiosa* (*Xf*) is a phytopathogenic bacterium with a repertoire of self-replicating genetic elements, including plasmids, pathogenicity islands, and prophages. These elements provide potential avenues for horizontal gene transfer both within and between species and have the ability to confer new virulence traits, including the ability to colonize new host plants. However, they can also serve as a ‘footprint’ to type plasmid-bearing strains. Genome sequencing of several strains of *Xf* subsp. *fastidiosa* sequence type (ST) 1 from Mallorca Island, Spain, revealed the presence of a 38 kb plasmid (pXFAS_5235). In this study, we developed a PCR-based typing approach using primers targeting the *traC* gene to determine the presence of pXFAS_5235 plasmid or other plasmids carrying this gene in a world-wide collection of 65 strains *X. fastidiosa* from different subspecies and STs or in 226 plant samples naturally infected by the bacterium obtained from the different outbreaks of *Xf* in Spain. The *traC* gene was amplified only in the plant samples obtained from Mallorca Island infected by *Xf* subsp. *fastidiosa* ST1 and from all Spanish strains belonging to this ST. Maximum-likelihood phylogenetic tree of *tra*C revealed a close relatedness among Spanish and Californian strains carrying similar plasmids. Our results confirm previous studies, which suggested that a single introduction event of *Xf* subsp. *fastidiosa* ST1 occurred in the Balearic Islands. Further studies on the presence and role of plasmids in *Xf* strains belonging to the same or different subspecies and STs can provide important information in studies of epidemiology, ecology, and evolution of this plant pathogen.

## 1. Introduction

*Xylella fastidiosa* (*Xf*) is a xylem-inhabiting plant pathogenic bacterium, native from the Americas, which can infect more than 650 plant species that have been reported as hosts [1]. This bacterium causes a variety of diseases on crops of high economic importance, including grapevine (Pierce’s disease, PD), citrus (Citrus Variegated Chlorosis, CVC), coffee (Coffee Leaf Scorch, CLS), almond trees (Almond Leaf Scorch, ALS), and olive trees (Olive Quick Decline Syndrome, OQDS) [1]. Currently, three *Xf* subspecies have been officially described (*fastidiosa*, *multiplex,* and *pauca*) [2,3,4] and two additional ones have been proposed (*morus* and *sandyi*) [5,6], but based on genomic analyses are included in the subspecies *fastidiosa* [7]. To date, within the subspecies level, 89 sequence types (ST) have been described based on MultiLocus Sequence Typing analysis (MLST) of seven housekeeping genes (*cysG*, *gltT*, *holC*, *leuA*, *malF*, *nuoL,* and *petC*) (https://pubmlst.org/organisms/xylella-fastidiosa; accessed on 12 August 2022) [8,9].

In Europe, the detection of *Xf* in the Salento region in Italy in 2013, which is associated with a massive mortality of olive trees [10,11] heightened big concerns on the risk of its spread across the continent. As a result, mandatory surveys of EU member states [12,13] were conducted revealing that the bacterium was also established in Corsica and Provence-Alpes-Côte d’Azur in France in 2015 [14], the Balearic Islands and Alicante in Spain in 2016 and 2017 [15,16], and in Porto, Lisbon, and Algarve in Portugal in 2019 and 2021 [17]. 

Results of official surveys in Balearic Islands since 2016 have revealed that the *Xf* genetic diversity in the islands is the highest within the different outbreaks in Europe. Therefore, to date, three *Xf* subspecies and five STs have been detected in the Balearic Islands [18,19,20]: (i) *Xf* subsp. *fastidiosa* ST1, which causes PD and ALS in California and also in Spain, recorded only on Mallorca Island; (ii) a new ST of *Xf* subsp. *pauca* (ST80), which is found only on Ibiza Island, mainly on wild and cultivated olive trees (*Olea europaea*); and (iii) another novel ST of *Xf* subsp. *multiplex* (ST81), which is present in the Mallorca and Menorca islands affecting mainly almonds, cultivated and wild olives, and *Ficus carica*. In addition, there have been three single detections of *Xf* subsp. *multiplex* ST7 in Mallorca Island on *Prunus dulcis* and *Polygala myrtifolia*.

In a recent study, phylogenetic analysis based on core genomes showed that isolates from *Xf* subsp. *fastidiosa* ST1 and *Xf* subsp. *multiplex* ST81 in the Balearic Islands shared their most recent common ancestors with Californian *Xf* populations. These isolates from California were associated with PD and ALS in grapevines and almonds, respectively, [18,20]. Moreover, all of the genomes obtained from several strains of *Xf* subsp. *fastidiosa* ST1 isolated from grapes and almonds in Mallorca revealed the presence of a 38 kb plasmid (pXFAS_5235), with a high sequence similarity to the conjugative plasmid pXFAS01 from isolate M23 of the same subspecies and ST, causing ALSD in California [18,21,22]. Interestingly, a nearly identical plasmid was found in *Xf* subsp. *multiplex* strain RIV5 [23]. The occurrence of similar plasmids in different subspecies of *Xf* represents an evidence of horizontal gene transfer (HGT) between strains [23] and together with recombination are hypothesized as one of the forces in the emergence of new *Xf* strains, which are capable of colonizing novel hosts [24].

It is known that *Xf* possesses a repertoire of mobile genetic elements (MGE), including prophages, pathogenicity islands, and plasmids [24]. The presence of large indigenous plasmids is frequent in bacteria that interact with plants, and in some circumstances, they confer virulence traits to plant-pathogenic bacteria, such as the Ti plasmid of *Agrobacterium tumefaciens* [25]. Bacterial plasmids are extrachromosomal DNA sequences, which are capable of autonomous replication and are a major source of HGT among bacteria, contributing to their evolution and adaptation [24,26]. Plasmids can be classified as self-transmissible or conjugative if they code for their own set of mating pair formation (MPF) proteins, such as the type IV coupling proteins (T4CP) and VirB4, which are part of the type IV secretion system (T4SS), enabling bacteria to efficiently exchange genetic material and proteins [27]. Therefore, plasmids play a very important role in bacterial pathogenesis and evolution.

Plasmid comparative sequence analyses and studies of their presence and distribution among different *Xf* strains belonging to the same or different subspecies and STs can provide important information regarding adaptation mechanisms of *Xf* strains to their hosts, with application in future studies of epidemiology, ecology, and evolution of this plant pathogen. Therefore, in this study, we used a PCR-based approach to describe the incidence and distribution of the pXFAS_5235 plasmid on a collection of several *Xf* strains from different subspecies and STs isolated from different host plants, different regions worldwide, and DNA samples extracted from *Xf*-infected plant samples, which are obtained from several hosts from different outbreak areas in Spain. On the basis of the hypothesis that the introduction of *Xf* in the Balearic Islands may have occurred in the early 1990s with the import of almond varieties [18], the analysis of the presence of pXFAS_5235 plasmid on *Xf* Spanish populations may provide interesting epidemiological information concerning the existence of different introduction events or of potential adaptation of the strains after some decades, since their introduction or after hosts jump from almonds into other crops and plants from the natural environment [19]. The objectives of this study were: (i) To develop a PCR-based typing approach using primers targeting the *traC* gene to determine the presence of pXFAS_5235 plasmid or other plasmids carrying this gene in a world-wide collection of *X. fastidiosa* strains from different subspecies and STs. (ii) To use this PCR-based typing approach for testing the presence of pXFAS_5235 plasmid in plant samples naturally infected by the bacterium obtained from the different outbreaks of *Xf* in Spain. (iii) To support the hypothesis that a single introduction event of *Xf* subsp. *fastidiosa* ST1 occurred in the Balearic Islands.

## 2. Results

### 2.1. Sensitivity of the traC PCR-Based Plasmid Typing Protocol 

**Figure 1:**
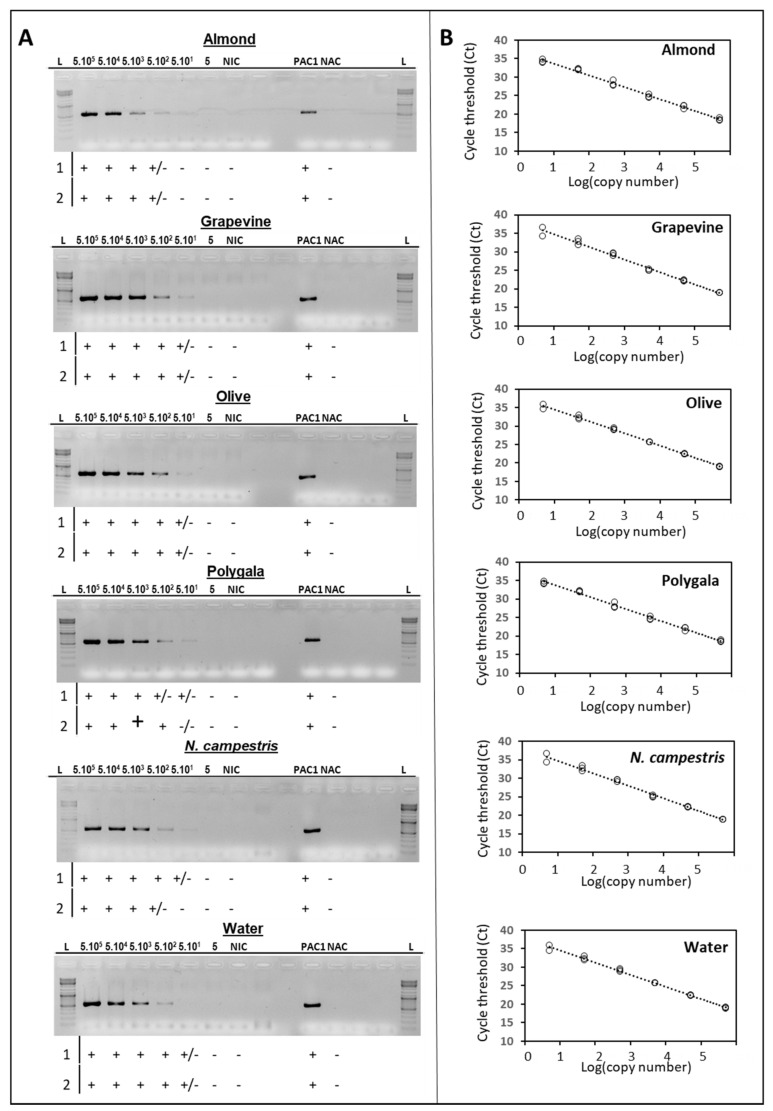
(**A**) Agarose gels showing the sensitivity of ND116-pRIV5-F1/ND117-pRIV5-R1 primer pair in the amplification of the *tra*C gene (expected amplicon size of 521 bp) using serial dilutions of *Xylella fastidiosa* (*Xf*) subspecies *fastidiosa* strain IVIA5235 DNA (from 5 × 10^5^ copies/µL; lanes 2 to 8) singly or mixed with host DNA (almond, grapevine, olive, polygala, *Neophilaenus campestris*, and water). NIC: Negative isolation control included plant insect DNA only. PAC: Positive amplification control contained boiled cells of IVIA5235 strain. NAC: Negative amplification control contained DNA from a *Xf* strain known not to harbor the *trac*C gene. L: 10 kb GeneRuler™ DNA Ladder Mix (Thermo Scientific™). Data shown below agarose gels summarize the results from two independent standard DNA curves (1 and 2): +L: Positive amplification; ±: Positive weak amplification; and −: No amplification. (**B**) Cycle thresholds obtained for Harper’s qPCR using the same DNA standard curves. Data correspond to three PCR amplifications from the serial DNA standard curve number 1, shown in (**A**).

The *traC* gene involved in DNA replication and transcription (locus AGC23499; previously D698_p2018) [23] was selected to type the presence of pXFAS_5235 plasmid. The sensitivity of the primer pair was evaluated on serial dilutions of *Xf* subspecies *fastidiosa* strain IVIA5235 DNA in different plant DNA backgrounds calibrated at an initial concentration of 5 × 10^5^ copies/µL. The latest and reproducible PCR amplification signal was obtained at 500 copies/µL and for all plant DNA backgrounds. However, for some of the two replications that were assessed, a very weak amplification could be observed at 50 copies/µL. However, it was not reproducible among the different experiments using independent standard curves and PCR replications, especially when using *Polygala* and insect (*N. campestris*) DNA backgrounds (Figure 1A). 

In addition, the same standard curves were assessed by qPCR using Harper’s test for comparison with the detection limit of the newly-developed *tra*C typing PCR. The Ct values for Harper’s qPCR that corresponded to 500 copies/µL ranged between 27.7 and 29.7 and those corresponding to 50 copies/µL ranged between 31.8 and 33.5, depending on the DNA background (host plant and insect DNA) and replication; however, the results were very reproducible (Figure 1B). These results indicated that for those plant DNA samples, which are naturally infected by *Xf* showing Harper’s qPCR Ct values higher or close to 28, there may be some chance of getting no amplification of the *tra*C gene using the ND116-pRIV5-F1/ND117-pRIV5-R1 primer pair, even if the *Xf* strain infecting the sample may harbor the pXFAS_5235 plasmid (Figure 1A). 

### 2.2. PCR-Based Plasmid Typing of Xylella fastidiosa Strains and Naturally-Infected Plant Samples

A total of five strains isolated in Mallorca Island belonging to *Xf* subsp. *fastidiosa* ST1 amplified the *traC* gene present in the pXFAS_5235 plasmid. The remaining Spanish strains analyzed belonging to *Xf* subsp. *multiplex* ST6 and ST81 or to *Xf* subsp. *pauca* ST80 showed no amplification of the *traC* gene (Table 1; Figure S1). Moreover, amplification was obtained from other strains of *Xf* subsp. *fastidiosa* ST1 (Temecula1, Temecula, M23, and CFBP8351), and two *Xf* subsp. *fastidiosa* ST2 strains (CFBP7970 and CFBP8082). *Xf* subsp. *multiplex* ST6 (strain Dixon) was the only strain from those tested belonging to subspecies *multiplex*, in which the *traC* gene was amplified. Furthermore, several *Xf* subsp. *pauca* strains from ST53 (CODIRO, De Donno, Salento-1, CFBP8495, and CFBP8429), ST73 (CFBP8498) and ST74 (CFBP8072 and CFBP8074), the recombinant strain CO33 of subsp. *sandyi/morus,* and strain CFBP8478 of *Xf* subsp. *sandyi* amplified the *traC* gene (Table 1; Figure S1). 

A total of 47 from 52 naturally-infected plant samples from Mallorca Island, which were previously characterized as infected by *Xf* subsp. *fastidiosa* ST1 with MLST analysis amplified the *tra*C gene, indicating the presence of pXFAS_5235 plasmid. On the other hand, we could not amplify the *tra*C gene in the remaining five plant samples. These samples showed a Ct > 30 for Harper’s qPCR test, which was over the detection limit of the ND116-pRIV5-F1 and ND117-pRIV5-R1 primer pair. Indeed, for those samples, it was necessary to perform the Nested-MLST analysis [28] to assign the *Xf* isolate infecting the sample at the subspecies and ST level. 

Nine of the 23 plant samples obtained from almond trunk rings, and previously shown as infected by *Xf* subsp. *fastidiosa* amplified the *tra*C gene, indicating the presence of pXFAS_5235 plasmid. From these positive samples, the DNA tree ring sample dated before 2004 was the oldest showing amplification (Table 2). For the remaining *Xf* subsp. *fastidiosa*-infected samples, in which there was no amplification of the *tra*C gene, the Ct values shown in Harper’s qPCR ranged between 25 and 36. 

Finally, as expected, 111 samples infected by *Xf* subsp. *multiplex* ST6 from Alicante (22 samples) and by *Xf* subsp. *multiplex* ST7 (3 samples) and ST81 (86 samples) from Mallorca and Menorca islands, as well as 40 samples infected by *Xf* subsp. *pauca* from Ibiza Island did not show amplification of the *tra*C gene (Table 2). 

### 2.3. Phylogenetic Analysis of traC Gene 

Alignments of the sequences for all *tra*C amplified products obtained from five Spanish strains of *Xf* subp. *fastidiosa* ST1 from Mallorca Island (Table 1) or from eight plant samples naturally infected by this strain type, including three samples from almond trunk rings (Table 2), indicated 100% homology among them and with strains Temecula1, TemeculaL, and M23 of *Xf* subp. *fastidiosa* ST1, as well as Dixon strain of *Xf* subp. *multiplex* ST6 (Figure S2). The *tra*C sequences from the remaining *Xf* strains included in this study presented up to five different sequences (Figure S2). As expected, in silico analysis of the consensus *tra*C gene sequence showed the best alignment score with the *tra*C sequence of the pXFAS_5235 plasmid (GenBank accession NZ_CP047172, in which ND116-pRIV5-F1 and ND117-pRIV5-R1 primer pair start at position 3639 and 3157, respectively) and the pXF-RIV5 and pXFAS01 plasmids present on *Xf* Dixon and Bakersfield-1 strains belonging to the subsp. *multiplex* and *fastidiosa,* respectively.

BLAST search at NCBI, using as query the *tra*C gene sequence of the pXFAS_5235 plasmid, matched with 58 drafts or completed whole-genomes of *Xf* with an identity between 95–99% and 99% query length coverage. As expected, the *tra*C gene sequence showed 100% homology with a total of 14 genomes available from Spanish strains of *Xf* subp. *fastidiosa* ST1 isolated from almond (7) and grape (7) from Mallorca Island. Interestingly, the *traC* gene was not exclusively found in *Xf* plasmids, but also on the chromosome of some *Xf* subsp. *pauca* strains from Brazil: 3124, U24d, J1a12, 9a5c, and Hib4. For strain PD7211 of *Xf* subsp. *pauca* ST73 intercepted in Netherlands, the *traC* gene was found in two different contigs: RRTZ01000001.1 and RRTZ01000002.1, both with 99% identity, but with 97.5% and 96.3% query length coverage, respectively.

Alignment of the *traC* gene of pXFAS-5235 against the RefSeq_genome and whole genome sequences databases revealed several polymorphisms between the *traC* gene sequence of strains belonging to the same subspecies *fastidiosa*, *multiplex*, and *pauca* from different STs and geographic origins (Figure S2). 

**Figure 2:**
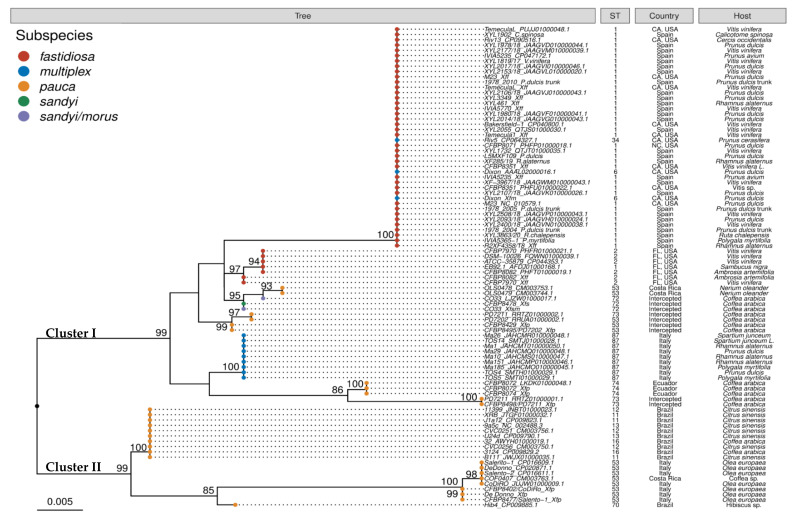
Maximum-likelihood phylogeny showing the genetic relationships among *traC* gene amplified from different *Xylella fastidiosa* strains and naturally-infected plants from this study and from drafts or finished whole-genomes of *Xf* present in the GenBank database. The numbers on the branches indicate the values of the bootstrap analyses.

Maximum-likelihood phylogenetic tree (Figure 2) was built showing the different *tra*C sequence types. The tree topology distinguished two main clusters (I and II) each with a bootstrap support of 99%, most of which were associated with different subspecies. Within cluster I, one of the main sub-clusters included all strains characterized as *Xf* subsp. *fastidiosa* ST1, with the exception of strain EB92.1 from Florida, USA, which was grouped in a second cluster with strains belonging to *Xf* subsp. *fastidiosa* ST2 (CFBP7970, CFBP8082, ATCC35879, and DSM_10026), which also originated from Florida. Moreover, this main cluster included *Xf* subsp. *multiplex* strains Dixon and RIV5. 

Interestingly, *tra*C sequences from strains belonging to *Xf* subspecies *pauca* ST53 appeared in the two main clusters and three different sub-clusters (Figure 2). Therefore, two strains of *Xf* subspecies *pauca* ST53 (OLS0478 and OLS0479) from Costa Rica grouped with strain CFBP8478 of *Xf* subsp. *sandyi* ST72 and the recombinant strain CO33 (93% bootstrap support), although their sequences presented some nucleotide polymorphisms (Figure S2). The next sub-cluster was constituted by the two strains of *Xf* subspecies *pauca* ST53 (CFBP8495/PD7202 and CFBP8429) isolated from *Coffea arabica* intercepted in Netherlands and France from plants from Costa Rica and grouped with strain CFBP8498/PD7211 of *Xf* subsp. *pauca* ST73. The last sub-cluster from cluster I included all *Xf* subsp. *multiplex* ST87 from the outbreak of Tuscany in Italy (TOS4, 5, 14 and MA1, 10, 26, 151, 185) grouped in an independent cluster closely related to strain CFBP8498/PD7211 belonging to *Xf* subsp. *pauca* ST73 from Costa Rica and strains CFBP8072 and CFBP8074 belonging to the *Xf* subspecies *pauca* ST74 from Ecuador. 

Cluster II was composed of three sub-clusters. The first one included several strains from diverse STs originated from Brazil and belonging to *Xf* subspecies *pauca* ST11 (XRB, J1a12, B111), ST12 (CVC0251, CVC0256), ST13 (U24d, 9a5c), and ST16 (32, 3124). A second sub-cluster grouped all *Xf* subspecies *pauca* ST53 from Italy and the coffee strain COF0407 from Costa Rica from the same ST53. The final sub-cluster close to this previous one included only Hib4 strain of *Xf* subspecies *pauca* ST70 from Brazil. 

## 3. Discussion

In this study, we have determined the presence of conjugative pXFAS_5235 plasmid in a collection of *Xf* strains and plant samples naturally infected by this bacterium obtained from the different outbreaks detected in Spain. In addition, throughout this work, we have assessed the incidence and distribution of the *traC* gene present in *Xf* strains belonging to different subspecies and STs from different regions of the world based on in silico and PCR-based amplification. In this case, we developed a PCR-based typing approach using the primer pair (ND116-pRIV5-F1 and ND117-pRIV5-R1) that targets the *tra*C gene within pXF-RIV5 plasmid from *Xf* subsp. *multiplex* strain RIV5 [23], whose sequence is nearly identical to pXFAS01 from *Xf* subsp. *fastidiosa* strain M23 and pXFAS_5235 plasmid present in the Spanish strain IVIA5235 [22].

The sensitivity of the primer pair was evaluated in different plant DNA backgrounds calibrated at an initial concentration of 5 × 10^5^ copies.µL^−1^. The fact that there are no differences between hosts in the sensitivity of the technique may be due to the use of BSA, which in other studies has been shown to compensate for polymerase inhibitors, and thus favor PCR efficiency [28]. In the same way, the gene could be amplified in naturally-infected samples from various hosts, where the lack of amplification was mainly related to a low concentration of bacterial cells (i.e., it was associated with high threshold values of Harper’s qPCR assay) [29]. 

Most of the *Xf* plant samples naturally infected by *Xf* subsp. *fastidiosa* ST1 amplified the *traC* gene, including DNA samples obtained from tree rings dated in 2004, which were previously shown as infected by *Xf* subsp. *fastidiosa*. The fact that in several tree ring samples known to be infected by *Xf* subsp. *fastidiosa* we could not achieve the *traC* could be due to the presence of PCR inhibitors, the type of sample processing (trunk vs. petiole) or a different DNA extraction protocol used for both plant tissues. An additional explanation, less plausible, may be that these rings were infected by a *Xf* subsp. *fastidiosa* strain not harboring the *tra*C gene or the pXFAS_5235 plasmid. This strain, for some reason, was not established in the island or may have been overlooked. 

Although the plasmid pXF64-Hb_ESVL from *Xf* subsp. *multiplex* ST6 showed a high sequence similarity to the conjugative plasmid pXF64-HB reported in *Xf* subsp. *pauca* ST70 [7,24,30], none of the samples characterized as infected by *Xf* subsp. *multiplex* ST6 from the Alicante focus (Valencian Community) amplified the *traC* gene. Since the role of *traC* is related to the family of conjugation proteins associated with the pilus structure [31], it would be interesting to study whether *Xf* subsp. *multiplex* ST6 conserves the complete set of potential conjugation genes to confirm whether this plasmid is capable of conjugative transfer, since this event plays a very important role in bacterial pathogenesis and evolution. However, pXF64-HB encodes several mating pair formation (MPF) proteins, including a MOBQ1 relaxase as well as one type IV coupling protein (T4CP) and one VirB4, which are key components of conjugative systems [24]. In addition, none of the Spanish strains or samples infected by *Xf* subsp. *pauca* ST80 from Ibiza Island showed amplification of the *traC* gene. Moreover, genome sequencing of the three isolates of *Xf* subsp *pauca* ST80 obtained from olive and almond trees in Ibiza Island have indicated that these strains do not harbor any plasmids (M.P. Velasco-Amo, L.F. Arias-Giraldo, and B.B. Landa, unpublished results). 

Identical *traC* sequence was amplified for all strains or plant samples naturally infected by *Xf* subsp. *fastidiosa* ST1 obtained from the Balearic Islands. In addition, its sequence showed 100% similarity to *traC* sequence from several strains from California, USA, including strains of different *Xf* subspecies, such as pXFAS01 in strains M23 and Bakersfield-1, pXFAS01-RIV13 in strain RIV13 of *Xf* subsp. *fastidiosa* ST1, and pXF879-41 in strain ATCC-35879 of *Xf* subsp. *fastidiosa* ST2; pXF31k6 in *Xf* subsp. *multiplex* ST6 strain Dixon, pXF-RIV5 in *Xf* subsp. *multiplex* ST34 strain RIV5 [23], placing those strains in the same node of the *tra*C ML phylogenetic tree (Figure 2). Similarly, Pierry et al. [24] found that ML phylogenetic trees of *X. fastidiosa* MOBP1 relaxases from plasmids were grouped as pXFAS_5235 with other plasmids from California, including pXF-RIV5, pXFAS01, and pXF31k6. These results support the evidence of previous studies that pointed out to a single introduction of *Xf* subsp. *fastidiosa* ST1 in the Balearic Islands with possible origin in the West Coast of the USA [16,18,19]. 

The *traC* gene was also amplified in strains of different *Xf* subsp. *pauca* from different STs (ST11, 12, 13, 16, 53, 70, 73, and 74), which were known to harbor a plasmid. However, there was a clear separation among the *traC* sequences found within the genome of *Xf* subsp. *pauca* strains, with those present in other subspecies, which agrees with the larger evolutionary distance of this subspecies as compared with subspecies *fastidiosa* and *multiplex*. Noticeably, up to three different *traC* sequences were found for strains belonging to *Xf* subsp. *pauca* ST53, which require further studies.

Interestingly, some *Xf* strains, whose complete genome is not available or its plasmid content is unknown, were amplified for *traC* gene, such as CFBP7970 and CFBP8082 from subsp. *fastidiosa*, Dixon from subsp. *multiplex,* CFBP8072 and CFBP8074 from subsp. *pauca*, and CFBP8478 from subsp *sandyi*. In these cases, an in-depth genomic analysis is required to determine whether this gene is conserved in the plasmid or whether it is inserted in the chromosome as with the strains 3124, U24d, J1a12, 9a5c, Hib4 from Brazil. Patterns of inter- and intrasubspecific homologous recombination between *Xf* subspecies *pauca* showed that these Brazilian strains have a high number of recombination events [32,33] (M.P. Velasco-Amo, L.F. and B.B. Landa, unpublished results). Plasmid integration into the bacterial chromosome is quite common [26] and at least one fifth of the genes in the pangenome of *Xf* originated from distant branches of the evolutionary tree, which were presumably acquired by HGT [34]. The biologically relevant scenarios, in which plasmid transfer might have occurred between different strains of *Xf*, would be in the xylem vessels of the host plants or the insect vectors. In some cases, *Xf* strains belonging to different subspecies have been isolated from the same host plants growing in the same location, although it is unclear whether this is a common occurrence [35]. This could explain why distant *Xf* strains belonging to different subspecies may harbor similar plasmids. 

To conclude, the new developed PCR-based typing approach to determine the presence of pXFAS_5235 plasmid and the ML phylogenetic analysis of *tra*C revealed a close relatedness among Spanish and Californian strains carrying similar plasmids. Our results confirm previous studies, which suggested that a single introduction event of *Xf* subsp. *fastidiosa* ST1 occurred in the Balearic Islands.

## 4. Materials and Methods

### 4.1. Bacterial and Plant Sample Sources and DNA Extraction Procedures

In this study, a total of 65 strains were used from the *Xf* collection at the Instituto de Agricultura Sostenible (IAS-CSIC), Córdoba, Spain belonging to subspecies *fastidiosa* (15 strains), *morus* (1 strain), *multiplex* (34 strains), *pauca* (11 strains), *sandyi* (3 strains), and a recombinant strain *pauca/sandyi* (Table 1). The strains isolated from the outbreaks in Alicante and Balearic islands in Spain were provided by different laboratories or acquired from the CFBP (Collection for Plant-associated Bacteria, INRAE, France). Strains were cultured in solid BCYE [36] or PD2 [37] solid media at 28 °C in the dark. After 7 to 12 days (depending on the strain), cells were collected for total DNA extraction using the DNeasy Plant Mini Kit (Quiagen). Total DNA were quantified in a ND-1000 spectrophotometer (Thermo Scientific™), and integrity was verified by 1% agarose gel electrophoresis.

A total of 203 plant samples naturally infected by *Xf* were obtained from different municipalities from Mallorca, Menorca, and Ibiza islands as well as from Alicante, Valencian Community, Spain, in the context of official monitoring surveys by the official diagnostic laboratories of Valencian Community and Balearic Islands in Spain (Table 2). Total plant DNA was extracted as described in the EPPO protocol [38] from leaf petioles or wood chip samples using the cetyltrimethylammonium bromide (CTAB)-based extraction or the EZNA HP Plant Mini kit (Omega-Biotek, Norcross, Georgia, USA), depending on the official laboratory providing the samples. DNA extracts were tested for the presence of *Xf* by Harper’s qPCR test with primers XF-F/XF-R and the dual-labeled probe XF-P [29], as described in [38]. Subspecies and ST assignation was performed using the conventional MLST analysis [8,9] or a newly developed Nested-MLST analysis [28] at the Instituto de Agricultura Sostenible IAS-CSIC, Córdoba, Spain.

In addition, a total of 23 DNA plants samples obtained from tree rings from a previous study were used [20]. These samples included tree rings dated back to 1995, in which the *Xf* presence was detected using Harper’s qPCR test and the subspecies of *Xf* was typed using a multiplex qPCR [39], conventional MLST analysis [8] or Nested-MLST analysis [28] (Table 2).

### 4.2. PCR-Based Plasmid Typing

Initially, the 38 kb sequence of pXF-RIV5 plasmid (GenBank accession JX548317) from *Xf* subsp. *multiplex* strain RIV5 [23,40] was used as a template to design primers with Primer3 software [41]. Primers were originally designed to test for the presence of pXF-RIV5 plasmid among the different *Xf* infected samples from the outbreaks in Corsica and PACA region in France (Denancé, N., *unpublished results*). For primer design, default software parameters were selected, including optimal expected size (20 bp), Tm (60 °C), and GC content (50%), as well as a size product between 500 and 1 kb. The primers ND116-pRIV5-F1 (5′-ACACACTCGCAAAGAACACC-3′) and ND117-pRIV5-R1 (5′-CACGCGGCTGATGAACATTA-3′) were selected, resulting in a yield of a 521 bp product. On the plasmid, ND116-pRIV5-F1 and ND117-pRIV5-R1 primer pair start at position 17,963 and 18,483, respectively, in *traC* gene involved in DNA replication and transcription (locus AGC23499; previously D698_p2018) [23]. The primers designed were checked by PCR for their ability to amplify the expected PCR product in *Xf* subsp. *fastidiosa* strain IVIA5235, once the availability of its genome revealed that this strain harbors the pXFAS_5235 plasmid, whose sequence is nearly identical to the pXF-RIV5 plasmid [21,22]. Moreover, the *Xf* subsp. *fastidiosa* strain M23 that harbors the pXFAS01 plasmid was used for confirmation. 

For typing of the *tra*C gene, PCR assays were performed in a final volume of 20 µL, including 1 µM of primers, 0.5 µg/µL of BSA (Molecular Biology Grade, New England Biolabs), 5X of Taq DNA Polymerase buffer, and 1 U of My Taq^TM^ DNA Polymerase (Bioline, London). The PCR was conducted in a T100 Thermal Cycler (BioRad, Hercules, CA, USA) using the following conditions: 94 °C for 3 min for initial denaturation; 34 cycles of 94 °C for 30 s, 60 °C for 30 s for annealing, and 72 °C for 45 s for strand elongation; at the end of all cycles, a step of 72 °C for 10 min for final elongation was performed. PCR products were evaluated by 1% agarose gel electrophoresis. DNA or boiled cells of *Xf* subsp. *fastidiosa* ST1 strain IVIA5235 harboring the pXFAS_5235 plasmid was used as a positive control. In addition, sterile ultrapure demineralized water was used as a blank and synthetic DNA of *Xf* subsp. *sandyi* (GenomiPhiTM DNA Amplification Kit; Amersham Biosciences Piscataway, NJ, USA) as a negative control.

Few representative PCR products were selected for Sanger sequencing using the same primers at the STAB Vida Lda sequencing facilities (Caparica, Portugal). Sequences have been deposited on GenBank.

### 4.3. Limit of Detection of the PCR-Based Plasmid Typing

To establish the limit of detection of the developed PCR test for typing the presence of plasmid pXFAS_5235 in plant DNA samples, two sets of DNA standard curves were obtained using 10-fold dilution series of *Xf* subsp. *fastidiosa* strain IVIA5235 DNA. For this purpose, *Xf* DNA from this strain was serially diluted in sterile ultrapure demineralized water as well as in a fixed background of host plant DNA (10 ng/µL) extracted from *Prunus dulcis* and *Olea europaea* wood chips, *Vitis vinifera* leaf petioles, *Polygala myrtifolia* leaf petioles or in a fixed background of insect DNA (10 ng/µL) from *Neophilaenus campestris* to obtain final concentrations starting from 5 × 10^5^ up to 5 copies of *Xf* DNA/µL. Each standard curve always included plant or insect DNA alone as negative control. Prior to the preparation of standard curves, *Xf* DNA was quantified in triplicate using the Quant-iT^TM^ PicoGreen^TM^ dsDNA Assay Kit (Thermo Scientific™) and a Tecan Safire microplate reader (Tecan Group, Männedorf, Switzerland) to ensure accurate DNA concentrations. 

Harper’s qPCR test [29] was performed on the same standard curves of *Xf* DNA in the different plants and insect DNA backgrounds to quantify the number of bacterial cells (copy number) and Ct values, at which the amplification of the pXFAS_5235 plasmid PCR signal was lost (i.e., limit of detection). The qPCR assays were performed on the thermal cycler LightCycler^®^ 480, Roche Applied Science, using 96-well plates (fits LightCycler^®^ 480 systems White, Landgraaf The Netherlands). The following thermal cycling program used was: 50 °C for 2 min, 95 °C for 10 min, then 45 cycles of two steps of 94 °C for 10 s and 62 °C for 40 s. The reaction mix was prepared in a final volume of 20 μL containing: iTaq™ Universal Probes Supermix (BioRad), 0.3 μM of each *Xf* forward and reverse primers (XF-F and XF-R, respectively), 0.1 μM of 6′FAM/BHQ-1 labeled probe (XF-P), 0.3 μg/μL of BSA (Molecular Biology Grade, New England Biolabs), and 2 μL of DNA sample. Three replicates of each concentration were tested simultaneously in the same run for each set of DNA standard curves. The data acquisitions and analyses were performed using LightCycler^®^ 480 Software, v1.5. Linear regressions of the natural logarithm of known concentrations of the target DNA vs. the Ct values were performed for each DNA standard curve. Ct values up to 35 were considered as a positive result. Ct values between 35 and 38 were considered as inconclusive. Therefore, the sample was repeated and samples with Ct values higher than 38 were considered as not *Xf* infected.

### 4.4. Sequencing and Phylogenetic Analysis of traC 

Sequence assembly for each sample was performed with Bionumerics v7.6 software package (Applied-Maths, Sint-Martens-Latem, Belgium). In addition, aligned sequences were submitted to BLAST searches for pairwise comparisons against the RefSeq_genome and whole genome sequences databases for *Xf* at GenBank. 

Alignments of all *tra*C sequences obtained in this study and those retrieved from GenBank were obtained using MAFFT [42], by selecting the “auto” mode for an appropriate alignment strategy. Subsequently, Spurious sequences or poorly aligned regions from the multiple sequence alignment were removed with trimAL v1.4.1 [43]. ModelTest-NG v0.1.6 [44] was executed with default parameters to select the appropriate substitution model for phylogenetic analyses. Based on the Bayesian information criterion (BIC) and the corrected Akaike information criterion (AIC), the TIM3ef + I + G substitution model was the best fit for our data out of the 80 DNA models tested. Finally, maximum-likelihood tree (ML) was computed using IQtree v2.2.0 [45] for DNA sequences with a 1000 bootstrap by the Ultrafast Bootstrap approximation approach (UFBoot) [46] and it was visualized with the R package ggtree v3.2.1 [47].

## Figures and Tables

**Table 1:** *Xylella fastidiosa* strain collection at the Institute of Sustainable Agriculture, Córdoba, Spain (IAS-CSIC), used in the study, which include different subspecies and sequence types (ST) and the results of the PCR-based typing of *tra*C gene using the ND116-pRIV5-F1/ND117-pRIV5-R1 primer pair.

Subspecies ^a^	ST ^a^	Strain	Origin	Host	*traC* Gene ^b^
*fastidiosa*	1	IVIA5235	Balearic Island, Spain	*Prunus avium*	+
		IVIA5770	Balearic Island, Spain	*Vitis vinifera*	+
		R2XF4358/18	Balearic Island, Spain	*Rhamnus alaternus*	+
		XYL461	Balearic Island, Spain	*Rhamnus alaternus*	+
		XYL3349	Balearic Island, Spain	*Prunus dulcis*	+
		CFBP8351	California, USA	*Vitis vinifera*	+
		Temecula1	California, USA	*Vitis vinifera*	+
		TemeculaL	California, USA	*Vitis vinifera*	+
		M23	California, USA	*Prunus dulcis*	+
*fastidiosa*	2	CFBP7970	Florida, USA	*Vitis vinifera*	+
		CFBP8082	Florida, USA	*Ambrosia artemifolia*	+
		WM1-1	Georgia, USA	*Vitis vinifera*	−
		CFBP7969	North Carolina, USA	*Vitis rotundifolia*	−
		CFBP8083	North Carolina, USA	*Vitis vinifera*	−
*fastidiosa*	75	CFBP8073	Mexico	*Coffea canephora*	−
*morus*	29	CFBP8084	Georgia, USA	*Morus alba*	−
*multiplex*	6	Dixon	California, USA	*Prunus dulcis*	+
		ESVL	Valencian Community, Spain	*Prunus dulcis*	−
		IVIA6902	Valencian Community, Spain	*Prunus dulcis*	−
		IAS-AXF212H7	Valencian Community, Spain	*Prunus dulcis*	−
		IAS-AXF235T1	Valencian Community, Spain	*Prunus dulcis*	−
		IAS-AXF235T10	Valencian Community, Spain	*Prunus dulcis*	−
		IAS-AXF64H11	Valencian Community, Spain	*Prunus dulcis*	−
		IAS-AXF64T12	Valencian Community, Spain	*Prunus dulcis*	−
		IAS-AXF64T13	Valencian Community, Spain	*Prunus dulcis*	−
		IAS-AXF64T14	Valencian Community, Spain	*Prunus dulcis*	−
		IVIA5901	Valencian Community, Spain	*Prunus dulcis*	−
		IVIA6586-2	Valencian Community, Spain	*Helicrysum italicum*	−
		IVIA6629	Valencian Community, Spain	*Rhamnus alaternus*	−
		CFBP8417	Corsica, France	*Spartium junceum*	−
		CFBP8418	Corsica, France	*Spartium junceum*	−
*multiplex*	7	CFBP8416	Corsica, France	*Polygala myrtifolia*	−
		M12	California, USA	*Prunus dulcis*	−
		LM10	California, USA	*Olea europaea*	−
		RH1	California, USA	*Olea europaea*	−
*multiplex*	10	CFBP8070	Georgia, USA	*Prunus* sp.	−
*multiplex*	27	CFBP8075	California, USA	*Prunus* sp.	−
*multiplex*	41	CFBP8173	Georgia, USA	*Prunus saliciana*	−
		CFBP8068	Washington DC, USA	*Ulmus* sp.	−
*multiplex*	42	AlmaEm3	Georgia, USA	*Vaccinium* sp.	−
*multiplex*	43	BB08-1	Florida, USA	*Vaccinium corymbosum*	−
*multiplex*	51	CFBP8078	Florida, USA	*Vinca* sp.	−
*multiplex*	81	XYL1981/17	Balearic Islands, Spain	*Ficus carica*	−
		XYL1966/18	Balearic Islands, Spain	*Olea europaea*	−
		XYL468	Balearic Islands, Spain	*Olea europaea*	−
		XYL466/19	Balearic Islands, Spain	*Olea europaea*	−
		XF3348	Balearic Islands, Spain	*Prunus dulcis*	−
		XYL1752/17	Balearic Islands, Spain	*Prunus dulcis*	−
		Santa29b	Balearic Islands, Spain	*Santolina chamaecyparissus*	−
		Fillmore	California, USA	*Olea europaea*	−
*pauca*	53	DeDonno	Apulia, Italy	*Olea europaea*	+
		CFBP8477/Salento-1	Apulia, Italy	*Olea europaea*	+
		CFBP8402/CoDiRo	Apulia, Italy	*Olea europaea*	+
		CFBP8495/PD7202	Intercepted, Costa Rica ^c^	*Coffea arabica*	+
		CFBP8429	Intercepted, unknown ^c^	*Coffea arabica*	+
*pauca*	73	CFBP8498/PD7211	Intercepted, Costa Rica ^c^	*Coffea arabica*	+
*pauca*	74	CFBP8072	Ecuador	*Coffea arabica*	+
		CFBP8074	Ecuador	*Coffea arabica*	+
*pauca*	80	XYL1961	Balearic Island, Spain	*Olea europaea*	−
		IAS-XYL1513-1	Balearic Island, Spain	*Prunus dulcis*	−
		IAS-XYL1518	Balearic Island, Spain	*Prunus dulcis*	−
*sandyi*	5	Ann-1	California, USA	*Nerium oleander*	−
*sandyi*	72	CFBP8478	Intercepted, Costa Rica ^c^	*Coffea arabica*	+
*sandyi*	76	CFBP8356	Intercepted, Costa Rica ^c^	*Coffea arabica*	−
*sandyi/morus*	72	CO33	Intercepted, Costa Rica ^c^	*Coffea arabica*	+

^a^ Subspecies and sequence type (ST) were determined by MLST analysis or by BLAST search of whole genome against the *Xylella fastidiosa* MLST database (https://pubmlst.org/xfastidiosa/). ^b^ Presence of *traC* gene by PCR-based plasmid typing was performed using ND116-pRIV5-F1 and ND117-pRIV5-R1 primers, pairs developed in this study. Positive and negative amplifications are represented as + or −, respectively. ^c^ Strains were isolated on the countries indicated from *Xf*-positive intercepted plants. Strains CFBP8495 and CFBP8498 were intercepted in Netherlands from plants from Costa Rica CO33 was intercepted in Italy from plants from Costa Rica. Strains CFBP8478 and CFBP8356 were intercepted in France from plants from Costa Rica, and CFBP8429 were intercepted in France from plants of unknown origin.

**Table 2:** Plant samples used in the study naturally infected by *X. fastidiosa*, with the information of host, geographical origin, and results of amplification of *tra*C gene, which was used as an indicator of the presence of pXFAS_5235 plasmid.

Subspecies ^a^	ST ^a^	Origin	Host ^b^	Ct ^c^	Number of Samples ^d^	*traC* Gene ^e^
*fastidiosa*	1	Mallorca	*Calicotome spinosa*	23, 27	2	2
*fastidiosa*	1	Mallorca	*Genista lucida*	20	1	1
*fastidiosa*	1	Mallorca	*Juglans regia*	29	1	1
*fastidiosa*	1	Mallorca	*Polygala myrtifolia*	23–28	4	4
*fastidiosa*	1	Mallorca	*Prunus dulcis*	21–31	23	19
*fastidiosa*	1	Mallorca	*Prunus dulcis* TR < 2004	25	2	2
*fastidiosa*	1	Mallorca	*Prunus dulcis* TR 2005–2009	22	2	2
*fastidiosa*	1	Mallorca	*Prunus dulcis* TR 2010–2017	22	5	5
*fastidiosa*	1	Mallorca	*Prunus dulcis* TR 1996–2015	25–26	14	0
*fastidiosa*	1	Mallorca	*Rhamnus alaternus*	20–27	8	8
*fastidiosa*	1	Mallorca	*Ruta chalepensis*	17–27	3	3
*fastidiosa*	1	Mallorca	*Teucrium capitatum*	33	1	0
*fastidiosa*	1	Mallorca	*Vitis vinifera*	21–25	9	9
*multiplex*	6	Alicante	*Prunus dulcis*	18–29	22	0
*multiplex*	7	Mallorca	*Polygala myrtifolia*	28	1	0
*multiplex*	7	Mallorca	*Prunus dulcis*	24, np	2	0
*multiplex*	81	Mallorca	*Acacia saligna*	29	1	0
*multiplex*	81	Mallorca	*Calicotome spinosa*	25	1	0
*multiplex*	81	Mallorca	*Cistus albidus*	33, 34	2	0
*multiplex*	81	Mallorca	*Ficus carica*	26–32	4	0
*multiplex*	81	Mallorca	*Fraxinus angustifolia*	24–30	9	0
*multiplex*	81	Mallorca	*Genista valdes-bermejoi*	24	1	0
*multiplex*	81	Mallorca	*Helichrysum stoechas*	28	1	0
*multiplex*	81	Mallorca	*Lavandula dentata*	25, 28	2	0
*multiplex*	81	Mallorca	*Olea europaea*	20–33	22	0
*multiplex*	81	Mallorca	*Phagnalon saxatile*	31	1	0
*multiplex*	81	Mallorca	*Phillyrea angustifolia*	32	1	0
*multiplex*	81	Mallorca	*Polygala myrtifolia*	22	1	0
*multiplex*	81	Mallorca	*Prunus dulcis*	25, 26	2	0
*multiplex*	81	Mallorca	*Rhamnus alaternus*	27	2	0
*multiplex*	81	Mallorca	*Rosmarinus officinalis*	33	1	0
*multiplex*	81	Mallorca	*Salvia officinalis*	23	1	0
*multiplex*	81	Mallorca	*Santolina chamaecyparissus*	20, 22	2	0
*multiplex*	81	Mallorca	*Spartium junceum*	21, 24	2	0
*multiplex*	81	Menorca	*Cistus albidus*	25, 26	2	0
*multiplex*	81	Menorca	*Clematis cirrhosa*	30	1	0
*multiplex*	81	Menorca	*Ficus carica*	28	1	0
*multiplex*	81	Menorca	*Helichrysum stoechas*	21, 27	2	0
*multiplex*	81	Menorca	*Olea europaea*	24–30	11	0
*multiplex*	81	Menorca	*Phlomis italica*	25, 27	2	0
*multiplex*	81	Menorca	*Rhamnus alaternus*	24, 35	2	0
*multiplex*	81	Menorca	*Rosmarinus officinalis*	26, 29	2	0
*multiplex*	81	Menorca	*Santolina chamaecyparissus*	24–31	5	0
*multiplex*	81	Menorca	*Santolina magonica*	29	1	0
*multiplex*	81	Menorca	*Vitex agnus-castus*	31	1	0
*pauca*	80	Ibiza	*Acacia saligna*	23, 24	2	0
*pauca*	80	Ibiza	*Cistus albidus*	25–30	3	0
*pauca*	80	Ibiza	*Genista hirsuta*	22	2	0
*pauca*	80	Ibiza	*Lavandula angustifolia*	23	1	0
*pauca*	80	Ibiza	*Lavandula dentata*	29	1	0
*pauca*	80	Ibiza	*Olea europaea*	24–32	15	0
*pauca*	80	Ibiza	*Polygala myrtifolia*	28, 31	2	0
*pauca*	80	Ibiza	*Prunus dulcis*	29–33	9	0
*pauca*	80	Ibiza	*Rosmarinus officinales*	29, np	3	0
*pauca*	80	Ibiza	*Ulex parviflorus*	21–25	2	0

^a^ Subspecies and sequence type (ST) were determined by MLST [8] or Nested-MLST [28] analysis. ^b^ All of the samples were obtained from fresh leaf petioles or stem portions of all analyzed hosts; except for samples of *Prunus dulcis* obtained from tree rings (TR) from a previous study [20]. ^c^ Ct: Cycle threshold value obtained for *Xf* amplification on those samples using Harper’s qPCR test [29]. Np: Not performed. ^d^ Number of samples analyzed for each host plant sampled during different years (2018–2022) and locations in official surveys. ^e^ Number of samples showing a positive amplification for *tra*C gene by the PCR-based plasmid typing with ND116-pRIV5-F1 and ND117-pRIV5-R1 primer pair.

## Data Availability

Data sharing is not applicable to this article. All data is contained in the article or as supplementary files.

## Supplementary Materials

The following supporting information can be downloaded at: https://www.mdpi.com/2223-7747/11/12/1562/s1. Figure S1: Agarose gels showing *traC* gene amplification for selected *X. fastidiosa* strains from the collection at the Instituto de Agricultura Sostenible (IAS-CSIC) using ND116-pRIV5-F1/ND117-pRIV5-R1 primer pair (expected amplicon size of 521 bp). PAC: Positive amplification control contained boiled cells of IVIA5235 strain. NAC: Negative amplification control contained DNA from a *Xf* strain known not to harbor the *traC* gene. L: 10 kb GeneRuler™ DNA Ladder Mix (Thermo Scientific™); Figure S2: Alignments of all *traC* sequences obtained in this study and those retrieved from GenBank using MAFFT. Single nucleotide polymorphisms are indicated in relation to the consensus sequence. The subspecies of the Xf strain is indicated between brackets (F: *fastidiosa*, M: *multiplex*, P: *pauca*, M: *morus*, S: *sandyi*, S/M: *sandyi/morus*).
