# Correction: Velasco-Amo et al. Use of *traC* Gene to Type the Incidence and Distribution of pXFAS_5235 Plasmid-Bearing Strains of *Xylella fastidiosa* subsp. *fastidiosa* ST1 in Spain. *Plants* 2022, *11*, 1562

**DOI:** 10.3390/plants13020200

**Published:** 2024-01-11

**Authors:** María Pilar Velasco-Amo, Luis F. Arias-Giraldo, Concepción Olivares-García, Nicolás Denancé, Marie-Agnès Jacques, Blanca B. Landa

**Affiliations:** 1 Institute for Sustainable Agriculture (IAS), Spanish National Research Council (CSIC), 14004 Córdoba, Spain; lfarias@ias.csic.es (L.F.A.-G.); colivares@ias.csic.es (C.O.-G.); 2 Groupe d’Étude et de controle des Variétes Et des Semences GEVES, CEDEX, F-49071 Beaucouzé, France; nicolas.denance@geves.fr; 3 University of Angers, Institut Agro, INRAE, IRHS, SFR QUASAV, F-49000 Angers, France; marie-agnes.jacques@inrae.fr

In the original publication [1], the information contained in Table 1 concerning the Sequence Type (ST) and host of some of the *Xylella fastidiosa* strains was not correct. *Xylella fastidiosa* subsp. *multiplex* strains RH1 and LM10 belong to ST 7 instead of ST 6. *Xylella fastidiosa* subspecies *multiplex* Fillmore belongs to ST 81 instead of ST 6. Regarding to *Xylella fastidiosa*, subsp. *multiplex* ST 6 IVIA6586-2 was isolated from *Helicrysum italicum*, and IVIA6629 was isolated from *Rhamnus alaternus* instead of *Prunus dulcis*, as it is said in the original table. This mistake does not affect the results or information provided in the main body of the manuscript.

The authors state that the scientific conclusions are unaffected. This correction was approved by the Academic Editor. The original publication has also been updated.

## Figures and Tables

**Table 1:** *Xylella fastidiosa* strain collection at the Institute of Sustainable Agriculture, Córdoba, Spain (IAS-CSIC), used in the study, which include different subspecies and sequence types (ST) and the results of the PCR-based typing of *traC* gene using the ND116-pRIV5-F1/ND117-pRIV5-R1 primer pair.

Subspecies ^a^	ST ^a^	Strain	Origin	Host	*traC* Gene ^b^
*fastidiosa*	1	IVIA5235	Balearic Island, Spain	*Prunus avium*	+
		IVIA5770	Balearic Island, Spain	*Vitis vinifera*	+
		R2XF4358/18	Balearic Island, Spain	*Rhamnus alaternus*	+
		XYL461	Balearic Island, Spain	*Rhamnus alaternus*	+
		XYL3349	Balearic Island, Spain	*Prunus dulcis*	+
		CFBP8351	California, USA	*Vitis vinifera*	+
		Temecula1	California, USA	*Vitis vinifera*	+
		TemeculaL	California, USA	*Vitis vinifera*	+
		M23	California, USA	*Prunus dulcis*	+
*fastidiosa*	2	CFBP7970	Florida, USA	*Vitis vinifera*	+
		CFBP8082	Florida, USA	*Ambrosia artemifolia*	+
		WM1-1	Georgia, USA	*Vitis vinifera*	−
		CFBP7969	North Carolina, USA	*Vitis rotundifolia*	−
		CFBP8083	North Carolina, USA	*Vitis vinifera*	−
*fastidiosa*	75	CFBP8073	Mexico	*Coffea canephora*	−
*morus*	29	CFBP8084	Georgia, USA	*Morus alba*	−
*multiplex*	6	Dixon	California, USA	*Prunus dulcis*	+
		ESVL	Valencian Community, Spain	*Prunus dulcis*	−
		IVIA6902	Valencian Community, Spain	*Prunus dulcis*	−
		IAS-AXF212H7	Valencian Community, Spain	*Prunus dulcis*	−
		IAS-AXF235T1	Valencian Community, Spain	*Prunus dulcis*	−
		IAS-AXF235T10	Valencian Community, Spain	*Prunus dulcis*	−
		IAS-AXF64H11	Valencian Community, Spain	*Prunus dulcis*	−
		IAS-AXF64T12	Valencian Community, Spain	*Prunus dulcis*	−
		IAS-AXF64T13	Valencian Community, Spain	*Prunus dulcis*	−
		IAS-AXF64T14	Valencian Community, Spain	*Prunus dulcis*	−
		IVIA5901	Valencian Community, Spain	*Prunus dulcis*	−
		IVIA6586-2	Valencian Community, Spain	*Helicrysum italicum*	−
		IVIA6629	Valencian Community, Spain	*Rhamnus alaternus*	−
		CFBP8417	Corsica, France	*Spartium junceum*	−
		CFBP8418	Corsica, France	*Spartium junceum*	−
*multiplex*	7	CFBP8416	Corsica, France	*Polygala myrtifolia*	−
		M12	California, USA	*Prunus dulcis*	−
		LM10	California, USA	*Olea europaea*	−
		RH1	California, USA	*Olea europaea*	−
*multiplex*	10	CFBP8070	Georgia, USA	*Prunus* sp.	−
*multiplex*	27	CFBP8075	California, USA	*Prunus* sp.	−
*multiplex*	41	CFBP8173	Georgia, USA	*Prunus saliciana*	−
		CFBP8068	Washington DC, USA	*Ulmus* sp.	−
*multiplex*	42	AlmaEm3	Georgia, USA	*Vaccinium* sp.	−
*multiplex*	43	BB08-1	Florida, USA	*Vaccinium corymbosum*	−
*multiplex*	51	CFBP8078	Florida, USA	*Vinca* sp.	−
*multiplex*	81	XYL1981/17	Balearic Islands, Spain	*Ficus carica*	−
		XYL1966/18	Balearic Islands, Spain	*Olea europaea*	−
		XYL468	Balearic Islands, Spain	*Olea europaea*	−
		XYL466/19	Balearic Islands, Spain	*Olea europaea*	−
		XF3348	Balearic Islands, Spain	*Prunus dulcis*	−
		XYL1752/17	Balearic Islands, Spain	*Prunus dulcis*	−
		Santa29b	Balearic Islands, Spain	*Santolina chamaecyparissus*	−
		Fillmore	California, USA	*Olea europaea*	−
*pauca*	53	DeDonno	Apulia, Italy	*Olea europaea*	+
		CFBP8477/Salento-1	Apulia, Italy	*Olea europaea*	+
		CFBP8402/CoDiRo	Apulia, Italy	*Olea europaea*	+
		CFBP8495/PD7202	Intercepted, Costa Rica ^c^	*Coffea arabica*	+
		CFBP8429	Intercepted, unknown ^c^	*Coffea arabica*	+
*pauca*	73	CFBP8498/PD7211	Intercepted, Costa Rica ^c^	*Coffea arabica*	+
*pauca*	74	CFBP8072	Ecuador	*Coffea arabica*	+
		CFBP8074	Ecuador	*Coffea arabica*	+
*pauca*	80	XYL1961	Balearic Island, Spain	*Olea europaea*	−
		IAS-XYL1513-1	Balearic Island, Spain	*Prunus dulcis*	−
		IAS-XYL1518	Balearic Island, Spain	*Prunus dulcis*	−
*sandyi*	5	Ann-1	California, USA	*Nerium oleander*	−
*sandyi*	72	CFBP8478	Intercepted, Costa Rica ^c^	*Coffea arabica*	+
*sandyi*	76	CFBP8356	Intercepted, Costa Rica ^c^	*Coffea arabica*	−
*sandyi/morus*	72	CO33	Intercepted, Costa Rica ^c^	*Coffea arabica*	+

^a^ Subspecies and sequence type (ST) were determined by MLST analysis or by BLAST search of whole genome against the *Xylella fastidiosa* MLST database (https://pubmlst.org/xfastidiosa/). ^b^ Presence of *traC* gene by PCR-based plasmid typing was performed using ND116-pRIV5-F1 and ND117-pRIV5-R1 primers, pairs developed in this study. Positive and negative amplifications are represented as + or −, respectively. ^c^ Strains were isolated on the countries indicated from *Xf*-positive intercepted plants. Strains CFBP8495 and CFBP8498 were intercepted in Netherlands from plants from Costa Rica CO33 was intercepted in Italy from plants from Costa Rica. Strains CFBP8478 and CFBP8356 were intercepted in France from plants from Costa Rica, and CFBP8429 were intercepted in France from plants of unknown origin.
