# Supplementary material for: Use of traC Gene to Type the Incidence and Distribution of pXFAS_5235 Plasmid-Bearing Strains of Xylella fastidiosa subsp. fastidiosa ST1 in Spain
Source: Plants (Basel). 2022 Jun 13;11(12):1562. doi: 10.3390/plants11121562 (PMC9228473; doi:10.3390/plants11121562)
Supplement: Supplementary file 1 [file plants-11-01562-s001.zip › Figure S2.pdf]

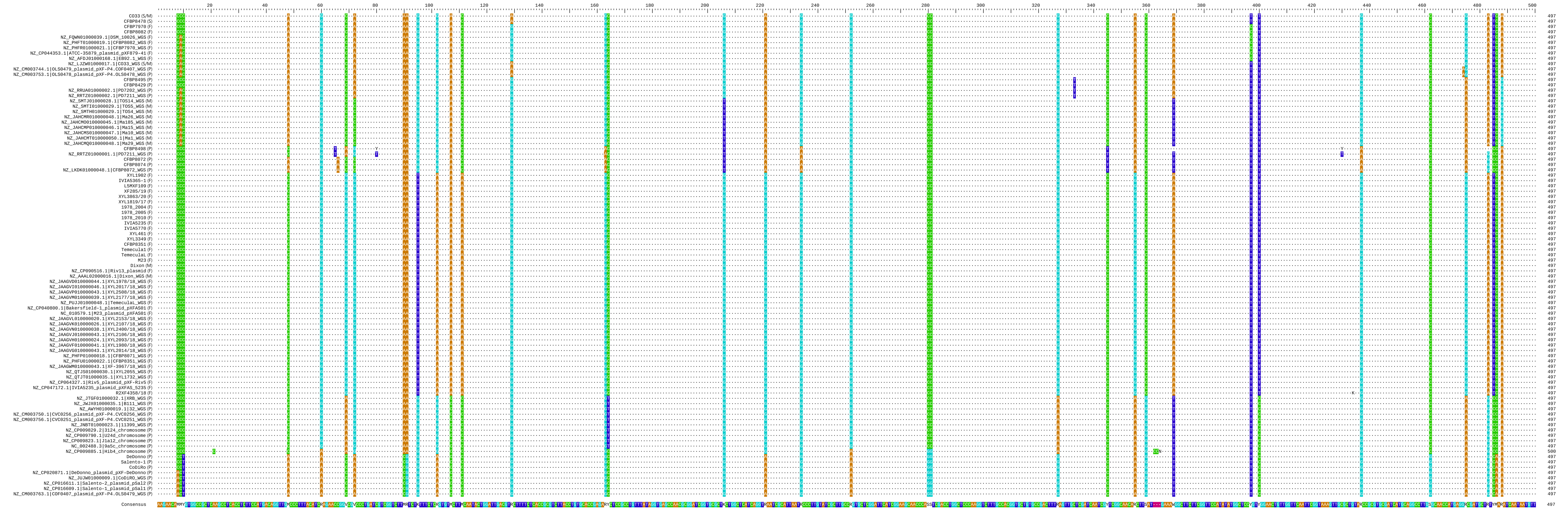
20 40 60 80 100 120 140 160 180 200 220 240 260 280 300 320 340 360 380 400 420 440 460 480 500
Consensus
